# Multiple-shower thromboembolism in an artificial mitral valve patient

**DOI:** 10.1186/1756-0500-6-4

**Published:** 2013-01-03

**Authors:** Ori Argov, Moshe Weintraub, Gideon Charach

**Affiliations:** 1 Department of Internal Medicine “C”, Tel Aviv Sourasky Medical Center, Weiztman street 6, Tel Aviv, 64239, Israel

## Abstract

**Background:**

Late acute left atrial thrombosis is a rare life-threatening complication that mostly appears with predisposing primary coagulopathy, such as Protein C, Protein S, antithrombin 3 deficiency, antiphospholipid syndrome or hyperhomocysteinemia. We present grave outcome due to lack of anticoagulation in a patient with artificial mitral valve.

**Case presentation:**

A 47-year-old male known to have an artificial valve was hospitalized in another hospital due to an acute illness. Anti-coagulation therapy was not provided during that hospitalization. He was transferred to our hospital due to lower limb weakness and diagnosed by us as having extensive emboli disease with complete occlusion of the distal aorta. Multiple infarcts were found in the abdominal organs and leg muscles. He suffered from multiple organ failure and eventually died.

**Conclusion:**

Neglecting the common practice of anticoagulation to a patient with a mechanical heart valve may, in rare cases, lead to immediate catastrophic event caused by shower thrombemboli with disseminated vascular occlusion from the left atrium to the abdominal aorta causing complete occlusion, spleen, kidney and muscle infarcts.

## Background

Patients with mechanical valves require anticoagulation therapy, usually delivered by oral administration of warfarin, a vitamin K antagonist. Failure to administer adequate therapy monitored by serial measurement of INR carries the risk of thrombus formation on the artificial valve whose dislodgment can cause catastrophic outcomes [1-3]. Routine INR measurement and adequate anticoagulation can be performed in hospitals, community clinics and even at home. Patients with mechanical heart valves are usually aware of the dangers of inadequate therapy. Those who are hospitalized are usually well monitored by daily blood tests and INR measurements, especially when they are known to be taking warfarin. There are many reasons for discontinuing warfarin therapy, including the inability to take oral medications due to an acute medical condition or a disturbance in the normal coagulation cascade (e.g., disseminated intravascular coagulation). Patients who can not take their regular warfarin therapy are put on unfractionated or low molecular weight heparin therapy. Warfarin should not, however, be stopped in a patient with a mechanical valve, and if there is no other option, replacement therapy should be instilled until it can be reintroduced.

## Case presentation

**Figure 1:**
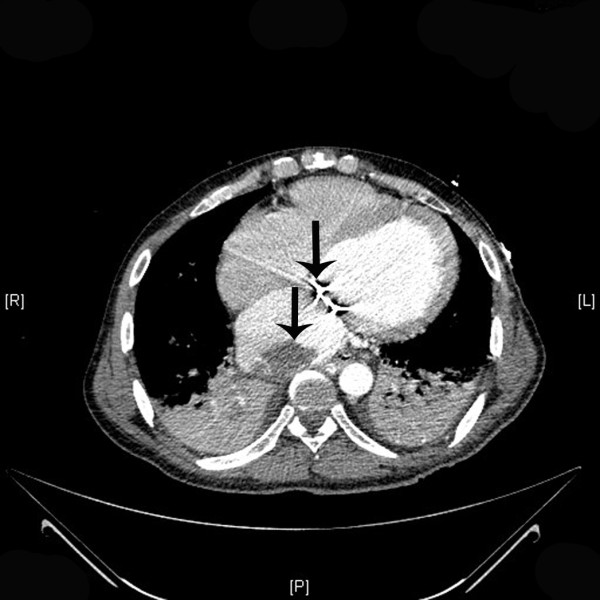
Transverse CT section at the heart level showing the artificial valve (upper arrow) and a thrombus in the left atrium (lower arrow).

**Figure 2:**
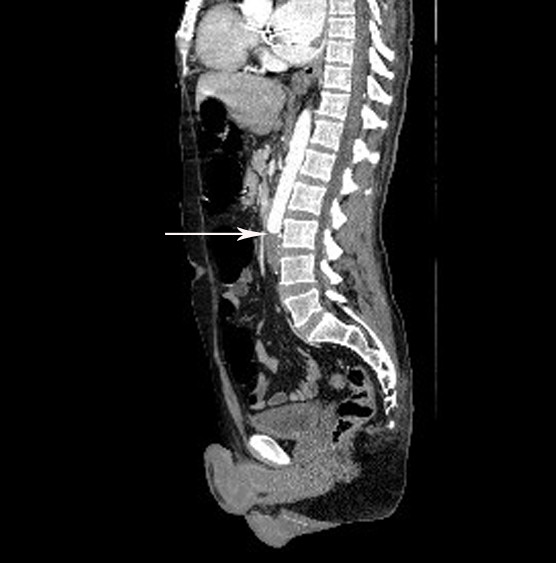
Sagittal CT section showing a thrombus in the distal aorta (arrow).

**Figure 3:**
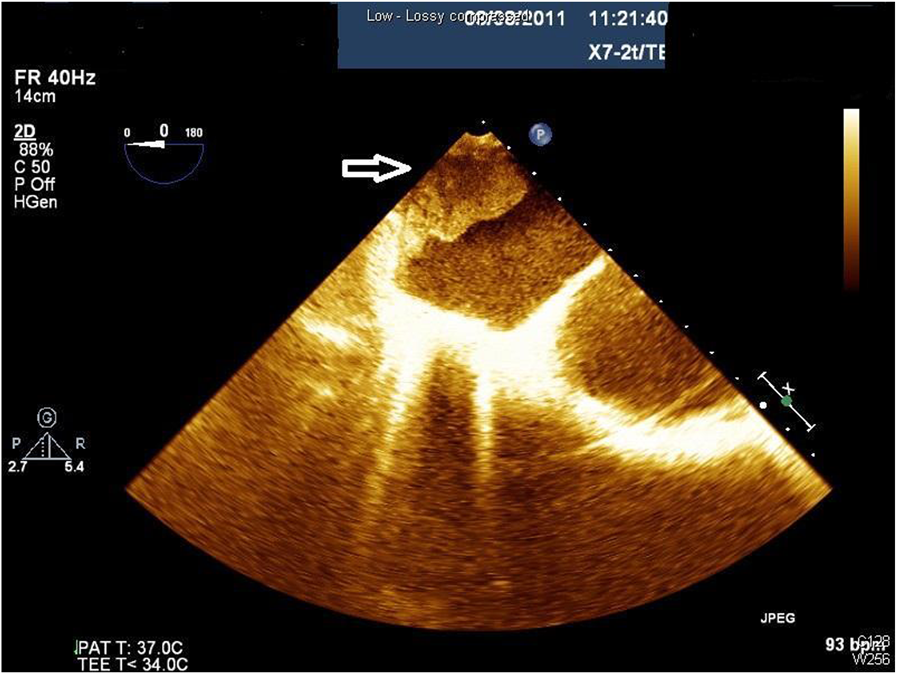
TEE showing a thrombus in the left atrium (arrow).

**Figure 4:**
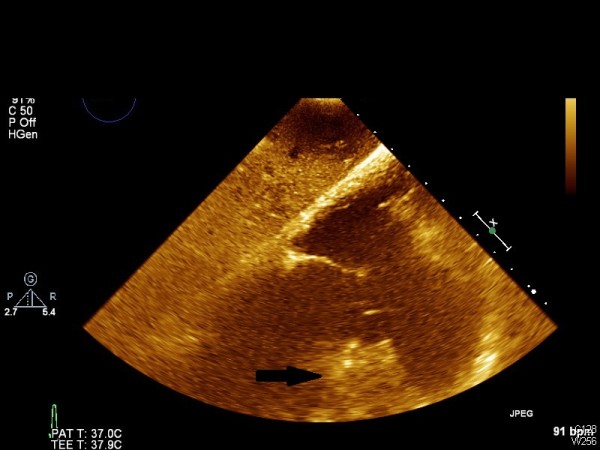
TTE showing a thrombus in the left atrium (arrow).

**Figure 5:**
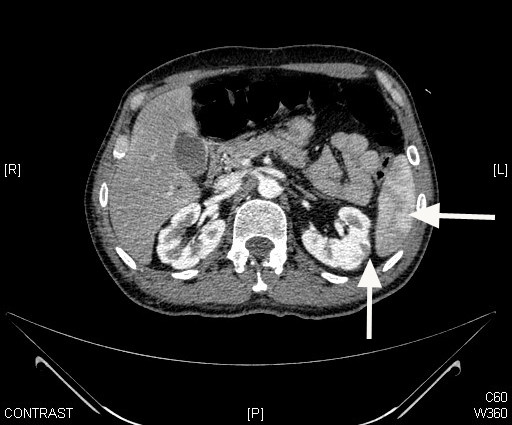
Axial CT scan showing infarcts in the spleen (right arrow) and the left kidney (left arrow).

**Figure 6:**
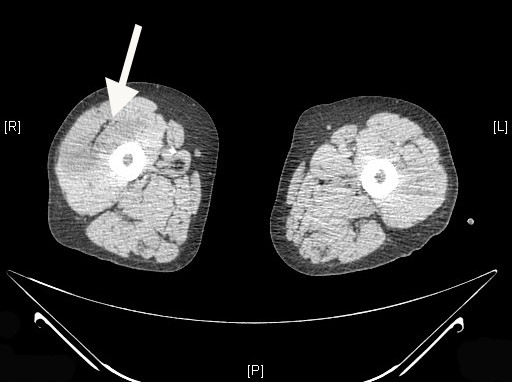
Axial CT scan showing a large infarct in the right quadriceps muscle (arrow).

A 47-year-old male was transferred to the emergency room from a hospital in the Gaza strip. The hospital’s medical summary described a patient who had an artificial mitral valve in place for the past 10 years. He had currently been hospitalized for abdominal pain and diarrhea and was transferred to our hospital for further investigation when he developed lower limb weakness. The patient was intubated upon arrival due to decreased consciousness and hypotension. Fluid and dopamine resuscitation was initiated. No pulse was palpated in the lower extremities which were pale and cold on physical examination. Blood tests were notable for renal failure and an INR of 1.0. Computerized tomographic (CT) angiography demonstrated an artificial mitral valve (Figure 1, right arrow) and a thrombus in the left atrium which was not attached to the mechanical valve (Figure 1, left arrow). A lower abdomen scan (Figure 2) showed a thrombus (arrow) completely obstructing the descending aorta, causing abrupt stopping of the progression of the intravenous contrast media (white). A bedside echocardiographic examination showed the thrombus in the left atrium (Figure 3, arrow, and Figure 4, arrow). Multiple spleen (Figure 5, right arrow), renal (Figure 5, lower arrow) and leg muscles infarcts (Figure 6, arrow) were also evident. The patient underwent urgent descending aorta thrombectomy which restored blood flow to the extremities. He was transferred to the intensive care unit where he received further fluid and amine resuscitation as well as hemodialysis. After few days of supporting therapy and anticoagulation the patient died of shock and multi organ failure caused by shower thromboembolism.

## Comment

We estimate that this patient underwent a prolong hospitalization due to a non- related disease of the gastrointestinal system. During this hospitalization, his regular warfarin treatment was either not administered or was administered in dosages that were inappropriately low for his special condition, hence the low INR level upon arrival. This caused the formation of a large thrombus attached to the artificial valve. This thrombus eventually dislodged and caused obstruction of the abdominal aorta. In this special case the patient was deprived of oral anticoagulant treatment before admission to our hospital and we encountered an exceptionally unique presentation of thromboemboli shower. This emphasizes the absolute need to switch to parenteral anticoagulation as early as possible in patients with mechanical valve at the mitral position.

## Conclusion

Anticoagulation therapy in a patient with an artificial valve is obligatory, even in those who are hospitalized due to an unrelated disease. Regular and frequent INR monitoring in such patients is advised as well. There are a number of reasons for inadequate anticoagulation therapy in hospitalized patients, among them the inability to take oral medications, failure to absorbed medications and medical conditions that require stopping the therapy (e.g. bleeding). The patient described in this report was unfortunately not provided adequate monitoring or treatment before he arrived to our hospital, and it was clear to us that the measures that we took by the time he came under our care were too late.

We report a rare catastrophic complication of multiple shower thromboembolism from the left atrium caused by complete occlusion of the abdominal aorta and multi organ infarcts in the spleen, kidney and muscle, although not causing a stuck valve. To our best knowledge, this is the first report of multiple embolism distribution with no thrombus in the patient’s artificial mitral valve.

## Consent

Written informed consent was obtained from the patient’s brother who escorted him along the hospitalization, for the publication of this case report and any accompanying images. A copy of the written consent is available for review by the Editor-in-Chief of this journal.

## Competing interests

The authors declare that they have no competing interests.

## Authors’ contributions

OA treated the patient, compiled and analyzed the patient’ s data and wrote the initial draft. GC reviewed the final manuscript and edited the coagulation aspect of the draft. All authors read and approved the final manuscript.

## References

[B1] Park JH Lee G Han JK Kim SJ Lee MM Inadvertent embolic obstruction of abdominal aorta from left atrial thrombus after percutaneous mitral valvuloplasty Cardiovasc Intervent Radiol 1990 13 6 351 353 10.1007/BF02578673 2126991

[B2] Okada K Yamashita C Okada M Ota T Ataka K Yoshida M Nohara H Azami T Yoshimura N Toyoda Y Acute left atrial thrombus causing cardiogenic shock following mitral valve replacement: report of a case Surg Today 1995 25 7 643 645 10.1007/BF00311440 7549278

[B3] Lee DH Jung TE Park SJ Acute post-cardiopulmonary bypass left atrial thrombosis after mitral valvuloplasty and left atrial thrombectomy J Cardiothorac Surg 2012 11 7:5 10.1186/1749-8090-7-5 PMC3269355 22236692

